# Descriptive epidemiology of anaemia among pregnant women initiating antenatal care in rural Northern Ghana

**DOI:** 10.4102/phcfm.v11i1.1892

**Published:** 2019-04-10

**Authors:** Engelbert A. Nonterah, Emmanuella Adomolga, Adadow Yidana, Juliana Kagura, Isaiah Agorinya, Emmanuel Y. Ayamba, Solomon Atindama, Michael B. Kaburise, Majeed Alhassan

**Affiliations:** 1 Navrongo Health Research Centre, Ghana Health Service, Navrongo, Ghana; 2 Navrongo War Memorial Hospital, Navrongo, Ghana; 3 Department of Community Health and Family Medicine, School of Medicine and Health Sciences, University for Development Studies, Tamale, Ghana; 4 Division of Epidemiology and Biostatistics, School of Public Health, University of the Witwatersrand, Johannesburg, South Africa; 5 Swiss TPH, University of Basel, Basel, Switzerland; 6 INDEPTH-Network, Mensah Wood Road, Accra, Ghana

## Abstract

**Background:**

Anaemia in pregnancy is associated with adverse obstetric outcomes. When detected early in pregnancy, it can be treated; however, information on its prevalence and associated factors is limited in rural Ghana.

**Aim:**

The aim of this study was to determine the prevalence and maternal factors associated with anaemia in pregnancy at first antenatal care (ANC) visits.

**Setting:**

The study was conducted in the Navrongo War Memorial Hospital, a secondary referral facility in the Kassena-Nankana district in rural northern Ghana.

**Methods:**

A retrospective analysis of antenatal clinic records of pregnant women collected from January to December 2014. All pregnant women initiating antenatal clinic, who had initial haemoglobin (Hb) levels measured, were included in the study. Logistic regression analyses were carried out to determine factors associated with anaemia at the initiation of ANC.

**Results:**

We analysed data from 506 women with median Hb of 11.1 g/dL (IQR 7.31–13.8). The median gestational age at booking was 14 weeks (5–36 weeks). The prevalence of anaemia was 42.7%, with 95% confidence interval (CI) [38.4–47.1], and was high among teenage mothers (52% [34.9–67.8]), mothers who booked in the third trimester (55% [33.6–74.7]) and grand multiparous women (58% [30.7–81.6]). Factors associated with anaemia included grand multiparity (odds ratio [OR] = 1.94 with 95% CI [1.58–2.46]), booking during the third trimester (OR = 2.06 [1.78–2.21]) and mother who were underweight compared to those with normal weight (OR = 3.17 [1.19–8.32]).

**Conclusion:**

Burden of anaemia in pregnancy is still high in rural northern Ghana. We advocate further strengthening of the primary health care system to improve early access to ANC delivery.

**Keywords:**

anaemia in pregnancy; booking visit; maternal and child health; Navrongo; rural; Ghana.

## Introduction

Anaemia in pregnancy is a major public health problem in lower middle income countries (LMICs).^1^ World Health Organization (WHO) estimates that Africa has the highest prevalence of anaemia in pregnancy.^1^ Various studies conducted in the African continent show significant variations in the prevalence of anaemia between countries.^2,3,4,5^ Ghana is among the countries in Africa with a high prevalence of anaemia in pregnancy.^6^ Rural–urban differences have also been reported with rural areas recording higher prevalence of anaemia.^4,6^

Defined by WHO as a haemoglobin (Hb) level of 11 g/dL and below or haematocrit level of less than 33%,^1^ anaemia in pregnancy is associated with adverse maternal and neonatal health outcomes such as miscarriages, stillbirths, intrauterine growth restriction, small for gestational age, perinatal anaemia and maternal mortality.^7,8,9,10^

The causes of anaemia in pregnancy are multi-factorial with iron, folate and other micronutrient deficiencies,^7,11^ reported in the literature as the most common causes. In LMIC such as Ghana, intestinal parasitic infections,^12^ malaria,^13,14^ HIV infection^15,16,17^ and haemoglobinopathies such as sickle cell anaemia and β-thalassaemias^18^ are major contributors to anaemia in pregnancy. Other intermediate obstetric causes of anaemia in pregnancy include teenage pregnancies, pregnancy among elderly women, very low body mass index (BMI) and more than five previous deliveries (grand multiparity).^6,19,20^ The relative contributions of geographical, socio-economic, religious, cultural and demographic factors and access to adequate health care services such as antenatal care (ANC) to anaemia in pregnancy have also been reported in the literature.^21^

Several policy guidelines in Ghana have sought to prevent and treat the causes of anaemia through strengthening of health systems such as improvements in maternal health care services offered at pre-, peri- and post-partum period (ANC, skilled deliveries and postnatal clinic [PNC]).^22^ The dividends have been countrywide improvements in current maternal health care indices, especially in rural areas where the Community-based Health Planning and Services (CHPS) initiative^23,24^ has been scaled up and expanded. Over 97% of pregnant women in Ghana have access to antenatal clinic services and 87% of women attended ANC at least four times^6^ as recommended by WHO^25^ and 74% proceeded to have deliveries conducted by skilled birth attendants.^6^ Iron and folate supplementation and use of antihelminthic to treat parasitic infection have also improved significantly among women attending ANC.^6^ Coverage of intermittent preventive treatment for malaria (IPT) using sulphadoxine-pyrimethamine and insecticide-treated nets (ITNs) has also improved significantly.^6,22,26^

Despite these interventions, the Ghana Demographic Health Survey reported high prevalence of anaemia in pregnancy in 2014.^6,27^ It is, however, not clear whether women are benefiting directly from these policy guidelines or not. It is also not clear whether pre-pregnancy anaemia, inherent maternal factors, poor client compliance to treatment or inappropriate treatment accounts for this persistently high burden of anaemia. However, early initiation of ANC has been proven to result in early detection and treatment of anaemia in pregnancy.^1,7,8,9,10^ Policy efforts must therefore be aimed at encouraging early initiation of ANC. Current knowledge on the prevalence of anaemia in pregnancy at the facility level is needed to inform this. Few studies have determined the prevalence and factors associated with anaemia among pregnant women in rural northern Ghana. To our knowledge, no study has reported on anaemia in pregnancy at the initiation of ANC care at the facility level. This study therefore seeks to determine the prevalence and maternal factors associated with anaemia among pregnant women at the first antenatal clinic visit in rural northern Ghana.

## Methods and design

### Study design

This was a cross-sectional study leveraged on secondary data from antenatal clinic records in the Navrongo War Memorial Hospital collected between January and December 2014.

### Study setting

This study was conducted among pregnant women initiating antenatal clinic care at the Navrongo War Memorial Hospital. The 123-bed facility is a referral hospital that serves the Kassena-Nankana East municipality, Kassena-Nankana West district and neighbouring communities from Burkina Faso. The hospital offers routine maternal and child health care services as well as comprehensive obstetric care services for an average population of 160 000 comprising 51% female.^28,29^ The area served by the hospital is predominantly rural (over 80%), with agriculture being the main source of employment.

Unpublished data from the hospital show an average annual outpatient department attendance of about 55 000 with an average of 10 000 admissions of which maternal-related conditions form part of the top ten reasons for admissions. The annual antenatal clinic attendance is about 2500 with roughly 1500 annual deliveries. Reproductive, maternal and child health profiles of the districts are similar to many rural districts in Ghana. The total fertility rate in 2002 was 4.5 which reduced to 3.5 in 2012.^28^ These rates are below national estimates. The crude birth rate for the district was 23.1 per 1000 people per year in June 2012, while the crude death rate was 11.1 per 1000 people per year for the same period.^28^ Neonatal mortality rate in 2012 was 14.3 per 1000 live births, while infant and under-five mortality rates were 30.4 and 56 per 1000 live births, respectively.^28^

### Data management

Antenatal clinic data are systematically documented using a structured Ghana Health Service data capture book.^6^ The information captured includes maternal demographic characteristics, parity, gestational age, history of previous pregnancies, weight, height measurements, Hb levels and relevant previous obstetric history. These data were extracted in duplicates by two trained research assistants to minimise errors in data entry.

Haemoglobin levels of all pregnant women are routinely measured at first ANC visits and at 36 weeks of gestation. All pregnant women, who attended ANC during the study period and had Hb levels measured in the hospital laboratory, were eligible for this study. For ease of comparison and to ensure a homogenous sample, pregnant women who had Hb levels measured outside the hospital facility were excluded from the analysis. The Hb levels were routinely measured to the nearest 0.1 g/dL using the SYSMEX KX-21N, Germany haematology analyser.

Weight and height were also measured using a calibrated standardised Seca GmH, (Hamburg, Germany) weighing scale and stadiometer. The documented information from the ANC records were extracted by two research assistants independently and entered into a designed Excel spreadsheet. The extracted data were then verified and confirmed by two physicians using confidential patient records from the medical records department. The entire data capture, entry and management processes were confidential. Participants were assigned codes and these were used instead of their names.

Age was categorised according to the Ghana Demographic Health Survey criteria into < 20 years, 20–34 years and 35–49 years; gestational age was categorised into first trimester (0–12 weeks), second trimester (13–27 weeks) and third trimester (28-delivery) and parity was categorised as nulliparity where index pregnancy is the first – multiparity as having greater than one and less than five previous pregnancies and grand multiparity as having greater than or equal to five previous pregnancies.^6^ Body mass index was categorised according the WHO recommendations into underweight < 18.5 kg/m^2^, normal weight = 18.5–24.9 kg/m^2^, overweight = 25–29.9 kg/m^2^ and obese > 30 kg/m^2^.^30^ Anaemia status of pregnant women was categorised into two mutually exclusive groups: anaemic as those with Hb < 11 g/dL and non-anaemic as those with Hb > 11 g/dL.^1^

### Statistical analysis

Relevant data from the Excel spreadsheet were exported into STATA version 14 (Statacorp LP, TX, United States [US]) where all analyses were conducted. The descriptive and inferential statistics are as presented in this article. Categorical variables are summarised using frequencies and proportions (%), while skewed continuous data are presented as median (interquartile range [IQR]). Prevalence (proportions) of anaemia with 95% confidence intervals (CIs) is presented for maternal characteristics. Differences in these proportions are examined using Pearson’s chi-squared (χ^2^) test. Factors associated with anaemia were assessed using multivariable logistic regression analysis. We initially assessed the independent association of maternal factors with anaemia at a statistical significance of *p* = 0.20. All factors significant at this level were incorporated in the final multivariable logistic regression analysis. A variance covariance approach was used. For all variables with more than two categories, a post-estimation test was used to determine the overall significance of the variable and a single *p*-value is subsequently presented in the regression output. The Hosmer–Lemeshow goodness-of-fit test was used to verify how well the data fitted in the final multivariable logistic regression model and a model with a *p* > 0.05 was considered to have a good fit. In addition, omitted-variable bias was assessed using the Ramsey regression specification error test. Factors associated with anaemia were those that had a *p* < 0.05.

### Ethical considerations

The study protocol was approved by the Department of Community Health and Family Medicine, School of Medicine and Health Sciences (SMHS) of the University for Development Studies, Tamale, Ghana. Additional written permission was obtained from the management of the Navrongo War Memorial Hospital to use the relevant data for the study. Secondary data were used and hence informed consent was waived, but the extracted data were subsequently de-identified to ensure confidentiality and to protect participants.

## Results

Records of a total of 650 pregnant women were extracted, but results are presented for 506 pregnant women who met the inclusion criteria for this study. Table 1 shows the basic characteristics of pregnant women initiating ANC at the Navrongo War Memorial Hospital in 2014.

**TABLE 1:** Basic characteristics of pregnant women initiating antenatal care at Navrongo War Memorial Hospital in 2014.

Maternal characteristics	Number†	Percentage
**Age categories in years**		
< 20 years	33	6.6
20–34 years	406	80.2
35–49 years	67	13.2
**Haemoglobin level in g/dL – median (IQR)**	11 (7.2–13.8)	-
**Anaemia status**		
Yes	216	42.7
No	290	57.3
**Parity**		
Nulliparity	144	28.4
Multiparity	350	69.2
Grand multiparity	12	2.4
**Gestational age – median (IQR)**	14 (5–36)	-
**Gestational age in trimesters**		
First trimester	238	47.0
Second trimester	248	49.0
Third trimester	20	4.0
**Use of ITN**		
Yes	280	55.3
No	226	44.7
**BMI categories**		
Underweight	27	5.3
Normal weight	293	57.9
Overweight	133	26.3
Obese	53	10.5

BMI, body mass index; IQR, interquartile range; ITN, insecticide-treated net.

† , *N* = 506.

Majority of the participants (80.2%) were between the 20 and 34 years age group, while 13.2% of participants were in the 35–49 years age group and 6.6% were less than 20 years of age (see Table 1). The participants had a median Hb level of 11.1 g/dL (IQR 7.2–13.8) with the median gestational age at booking being 14 weeks (IQR 5–36). The overall prevalence of anaemia in the study population was 42.7%, 95% CI (38.4–47.1).

Nearly half (49%) of the pregnant women attended their first ANC visit during the second trimester. More than half (55.3%) of the pregnant women were using ITNs. The median parity of pregnant women in this study was 1 (0–9), with the majority of women (69.2%) reporting between 1 and 5 previous pregnancies. Women presented for booking with a normal weight were 57.9%, while 1 in 10 women was obese and 5.3% were underweight (see Table 1).

Having more than five previous pregnancies (grand multiparous women) was associated with a higher prevalence of anaemia (58.3% with 95% CI [30.7–81.6]) compared with those who were pregnant for the first time (48.6% with 95% CI [40.5–56.8]). The prevalence of anaemia differed by gestation at booking (*p* = 0.045) and parity (*p* < 0.0001). Prevalence of anaemia did not differ according to use of ITN, and the differences observed for BMI were not statically significant (see Table 2).

**TABLE 2:** Prevalence of anaemia by maternal characteristics of pregnant women initiating antenatal care at Navrongo War Memorial Hospital in 2014.

Maternal characteristics	Total (*N*)	Yes – anaemia			No – anaemia		
		*n*	Prevalence (%)	95% CI	*n*	Prevalence (%)	95% CI
Parity***						
Nulliparity	144	70	48.6	40.5–56.8	74	51.4	43.2–59.5
Multiparity	350	139	39.7	34.7–45.0	211	60.3	55.0–65.3
Grand multiparity	12	7	58.3	30.7–81.6	5	41.7	18.4–69.3
**Use of ITNs**			
Yes	280	120	42.9	37.2–48.7	160	57.1	51.3–62.8
No	225	96	42.7	36.3–49.2	129	57.3	50.8–63.7
**BMI categories**						
Underweight	26	6	23.1	10.7–42.8	20	76.9	57.2–89.3
Normal weight	292	134	45.9	40.2–51.7	158	54.1	48.3–59.8
Overweight	132	54	40.9	32.8–49.5	78	59.1	50.5–67.2
Obese	52	20	38.5	26.3–52.3	32	61.5	47.7–73.7
Gestational age*							
First trimester	237	98	41.4	35.2–47.7	139	58.6	52.3–64.8
Second trimester	248	106	42.7	36.7–49.0	142	57.3	51.0–63.3
Third trimester	20	11	55.0	33.6–74.7	9	45.0	25.3–66.4

Note: Statistically significant differences in prevalence of anaemia by selected maternal characteristics are indicated by asterisks.

CI, confidence interval; ITNs, insecticide-treated nets; BMI, body mass index.

* , *p* < 0.05;

** , *p* < 0.01;

*** , *p* < 0.0001.

**FIGURE 1:**
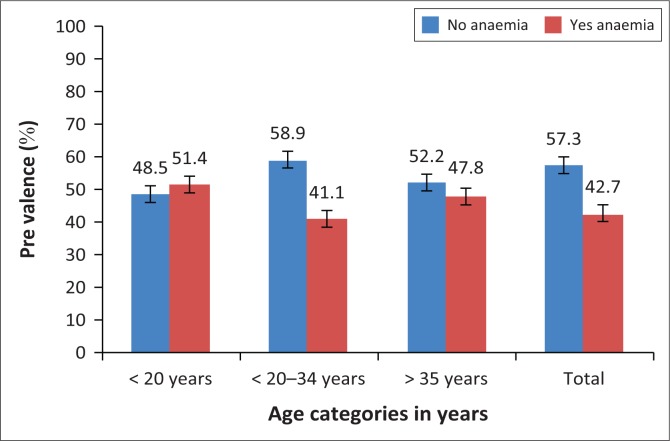
Prevalence of anaemia by age categories in years of pregnant women in Navrongo War Memorial Hospital in 2014.

The prevalence of anaemia also differed by maternal age (*p* = 0.002). Expectant mothers less than 20 years old were more likely to be anaemic (51.5% with 95% CI [34.9–67.8]) compared with those greater than 35 years (47.8% with 95% CI [36.1–59.7]) and those within the age group 20–35 years (41% with 95% CI [36.4–46.0]) (see Figure 1).

The study also determined maternal factors associated with anaemia in pregnancy for women initiating ANC in 2014, and these results are presented in Table 3. At the univariable analysis, factors associated with anaemia at booking were age of pregnant woman (*p* = 0.134), parity at booking (*p* = 0.106), gestational age at booking (*p* = 0.07) and BMI at booking (*p* = 0.031).

**TABLE 3:** Factors associated with anaemia among pregnant women at booking in Navrongo War Memorial Hospital in 2014.

Maternal characteristics	Unadjusted OR (95% CI)	*p*	Adjusted OR† (95% CI)	*p*
**Age categories in years**			
< 20 years	1.52 (0.74–3.09)	0.134	1.32 (0.62–2.85)	0.238
20–35 years	1	-	1	-
> 35 years	1.31 (0.78–2.19)	-	1.56 (0.89–2.73)	-
**Parity**			
Nulliparity	1.43 (0.97–2.12)	0.106	**1.50 (1.09–2.30)**	0.043
Multiparity	1	-	1	-
Grand multiparity	2.12 (0.66–6.83)	-	**1.94 (1.58–2.46)**	-
**Gestational age at booking**			
First trimester	0.94 (0.65–1.35)	0.070	**0.91 (0.62–0.99)**	0.005
Second trimester	1	-	1	-
Third trimester	1.64 (0.65–4.09)	-	**2.06 (1.78–2.21)**	-
**Use of ITNs**			
Yes	1	-	-	-
No	0.99 (0.69–1.41)	0.966	-	-
**BMI categories**			
Underweight	3.54 (1.10–4.25)	0.031	**3.17 (1.19–8.32)**	0.019
Normal weight	1	-	1	-
Overweight	0.82 (0.53–0.98)	-	**0.80 (0.39–0.97)**	-
Obese	0.74 (0.48–1.03)	-	**0.64 (0.34–0.89)**	-

Note: Data set in bold refers to factors associated with anaemia at *p* < 0.05.

OR, odds ratio; CI, confidence interval; ITN, insecticide-treated nets; BMI, body mass index.

† , Adjusted for variables in the table and reference value = 1.

After adjusting for these factors, we observed varied associations between parity at booking (*p* = 0.043), gestational age at booking (*p* = 0.005) and BMI (*p* = 0.019). Pregnant women presenting at booking for the first pregnancy (OR 1.50, 95% CI [1.09–2.30]) and grand multiparous women (OR 1.94, 95% CI [1.58–2.46]) were likely to present with anaemia compared with multiparous women. Participants who initiated ANC in the first 12 weeks of pregnancy had a 9% (OR 0.91, 95% CI [0.62–0.99]) reduction in risk of anaemia, while those who initiated ANC after 28 weeks of gestation had a twofold increase in risk of anaemia (OR 2.06, 95% CI [1.78–2.21]) compared with those who booked in the second trimester. Compared with normal weight, being underweight carried a greater risk of anaemia (OR 3.17, 95% CI [1.19–8.32]), while overweight (OR 0.80, 95% CI [0.39–0.97]) and obesity (OR 0.64, 95% CI [0.34–0.89]) were associated with 20% and 36% reduction in risk of anaemia, respectively (see Table 3).

## Discussion

We set out to determine the prevalence and maternal factors associated with anaemia among pregnant women initiating ANC care in the Navrongo War Memorial Hospital. We observed a high prevalence of anaemia among study participants. This high prevalence mirrors the prevalence reported by Ghana Demographic Health Survey, (GSS 2014)^6^ and studies from other settings that share similar profile as ours.^3,4,13^

The study showed that most women in Navrongo booked for ANC in their second trimester. This is consistent with findings in the 2014 Ghana DHS report^6^ and reflects the nationwide pattern by most pregnant women. Women in their teenage years were likely to present with anaemia compared with older mothers. Previous studies have reported similar findings.^31,32^ Those booking in their third trimester had the highest prevalence of anaemia, while those booking in the first trimester had the lowest prevalence. This presents a worrying phenomenon because such women are reported to present with adverse maternal and perinatal outcomes.^33^ Although other reproductive and past obstetric factors play a key role in pregnancy-related anaemia,^21^ early initiation of antenatal clinic attendance has potential beneficial effects for both mother and unborn baby.

The use of ITNs was generally high and was similar to previous estimates in the same setting,^34^ but did not differ between anaemic and non-anaemic participants. Further univariable and multivariable regression analysis did not find any association between ITN use and anaemia in pregnancy. This is contrary to previous randomised control trials which reported ITN use during pregnancy as an effective means of controlling malaria and by extension leads to a reduction in anaemia in pregnancy.^35^ Our study could not analyse the incidence of malaria among users and non-users of ITNs, hence it would be inconclusive to state clearly the impact of ITNs on anaemia in pregnancy in this study population. However, the existence of other effective forms of malaria prevention such as chemoprophylaxis with IPT, which was scaled up in the study area, could be a mitigating factor.

The maternal factors associated with anaemia in the cohort of pregnant women in Navrongo include parity, gestational age and BMI. There is paucity of data on the association between BMI and anaemia in both the general population and especially among pregnant women. Although most studies had attributed iron deficiency with overweight and obesity,^19,36^ other studies have reported the inverse.^37^ We observed that underweight compared with normal weight was associated with increased risk of anaemia. This is likely to be because of the fact that poor nutritional status is associated with underweight and low iron stores.^8^

We also report of an association between parity and anaemia in pregnancy, and this was similar to findings from a previous study in our study setting.^34^ Other cohort studies have also established that increasing parity confers a higher risk of anaemia in pregnancy.^20^ With increasing parity there is limited time for women to recover from previous pregnancy-related anaemia between successive pregnancies. This is likely to worsen the physiological anaemia encountered during pregnancy and therefore increase the risk of bleeding (haemorrhage) before, during and after delivery.^20,38^

Similar to our findings, other studies have reported a positive correlation between higher gestational age at booking for ANC and maternal anaemia^33^ which often leads to limited time for optimisation of Hb levels before delivery. In resource-limited settings, this is likely to result in adverse maternal and perinatal outcomes.

### Strengths

The study gives perspective to the burden of anaemia in a rural district hospital as well as the potential-associated maternal risk factors. It also serves as a benchmark for further research into the role of other factors that may contribute to understanding anaemia in pregnancy at initiation of ANC care. The study also gives a picture of the facility level prevalence of anaemia in rural northern Ghana. This study also raises potential questions for further research into the factors that prevent early initiation of ANC in a setting where there are many primary level health care facilities.

### Limitations

Despite these strengths there are several limitations to our study. Because of its cross-sectional nature, we are unable to establish cause of anaemia from the various maternal factors examined. As a retrospective analysis of hospital records, we were limited in the range of possible confounding variables that could influence our measured associations. These estimated associations could therefore be biased. These omitted variables include incidence of malaria and use of IPT, the socio-economic status of mothers, HIV status of mothers, pre-pregnancy Hb levels and pre-pregnancy BMI as pregnancy is associated with weight gain and dietary history during pregnancy.

## Conclusion

In this study we have established that a high prevalence of anaemia among younger maternal age group at the time of initiating ANC. We also observed that increasing parity at booking, increasing gestational age at booking and being underweight at booking were associated with anaemia among pregnant women.

We therefore advocate for the use of community-level primary health care facilities to encourage early initiation of ANC as this will lead to substantial reduction in maternal and perinatal outcomes.
